# Urban-development-induced Changes in the Diversity and Composition of the Soil Bacterial Community in Beijing

**DOI:** 10.1038/srep38811

**Published:** 2016-12-09

**Authors:** Bing Yan, Junsheng Li, Nengwen Xiao, Yue Qi, Gang Fu, Gaohui Liu, Mengping Qiao

**Affiliations:** 1 College of Water Sciences, Beijing Normal University, Beijing, China; 2 Chinese Research Academy of Environmental Sciences, Beijing, China; 3 School of Environment and Natural Resources, Renmin University of China, Beijing, China

## Abstract

Numerous studies have implicated urbanization as a major cause of loss of biodiversity. Most of them have focused on plants and animals, even though soil microorganisms make up a large proportion of that biodiversity. However, it is unclear how the soil bacterial community is affected by urban development. Here, paired-end Illumina sequencing of the 16 S rRNA gene at V4 region was performed to study the soil microbial community across Beijing’s built-up area. Proteobacteria, Acidobacteria, Bacteroidetes, Actinobacteria, Gemmatimonadetes, Verrucomicrobia, Planctomycetes, and Chloroflexi were the dominant phyla in all samples, but the relative abundance of these phyla differed significantly across these concentric zones. The diversity and composition of the soil bacterial community were found to be closely correlated with soil pH. Variance partitioning analysis suggested that urban ring roads contributed 5.95% of the bacterial community variation, and soil environmental factors explained 17.65% of the variation. The results of the current work indicate that urban development can alter the composition and diversity of the soil microbial community, and showed pH to be a key factor in the shaping of the composition of the soil bacterial community. Urban development did have a strong impact on the bacterial community of urban soil in Beijing.

Globally, urbanization is proceeding rapidly and now poses a major threat to biodiversity^1^,^2^,^3^,^4^ and it is a principal cause of biotic homogenization^5^,^6^. In the past few years, most studies of the effect of urbanization have focused on plants and animals^7^,^8^,^9^, even though microbial communities in soil are incredibly abundant and diverse^10^,^11^ and play important roles in biogeochemical cycles^12^ and nutrient cycles^13^,^14^. However, although the conservation of microbial biodiversity is receiving increasing amounts of attention^15^,^16^,^17^, the effect of urbanization on microorganisms remains largely unknown.

The diversity of microbes in soil is enormous and complex^10^,^18^, and many studies have attempted to determine how soil microbes might be influenced by biotic and abiotic factors. Identifying the dominant factors that affect the soil bacterial communities is crucial to understanding the diversity of the soil bacterial community. Various studies have generally considered soil pH to be an important factor in determining the distribution of soil bacterial communities^19^,^20^,^21^,^22^,^23^. Recent studies have shown bacterial diversity to be determined predominantly by soil pH^24^,^25^, but other soil parameters can also influence the composition and diversity of the bacterial community. Soil organic carbon and C/N ratios are here considered important influencers of soil microbial communities^26^,^27^,^28^. Soil nutrient availability has been found to have a significant impact on soil microbial communities^29^. Soil moisture^30^, and salinity^31^ have also been shown to be significantly correlated with the structure of soil microbial communities. Some studies have shown that climate change factors, including elevated CO_2_, temperature and precipitation, can influence the composition of soil microbial community in some specific ecosystems^32^,^33^,^34^,^35^. Yuan *et al*. stated that precipitation would be an important factor for changes of soil bacterial communities^36^. In this way, soil microbial communities can be influenced by a large number of factors, but the driving factor may be different in different ecosystems.

As described above, most soil microbial studies have been conducted in natural settings, but urban ecosystems are more complex than other ecosystems. Urbanization processes have been shown to affect soil ecosystems^37^,^38^. Urbanization is associated with a variety of effects on the physical and chemical properties of the soil, including the urban heat island effect^39^, pollution^40^, conversion to various forms of land use, and soil community changes^41^. These things can alter the structure and functionality of urban soil^42^. It is here speculated that these changes in soil chemical and physical properties may affect the soil microorganisms in urban areas.

The effect of urbanization on soil microbial communities has received relatively little attention^43^. Recently, Xu *et al*. suggested that urbanization affects the composition of bacterial community in urban park soils^44^. Ramirez *et al*. found there to be similar bacterial diversity between soil from Central Park and the global data set^45^. Barberán *et al*. also found that the microbes present in dust did not differ significantly between the areas within and outside of cities^46^. However, it remains unclear how urban development influences soil microbial communities. Urban soil microbial communities are also critical to urban ecosystem function and perhaps even human health^47^. These are an important source of airborne microbial diversity^48^. More studies are needed on this topic.

Specifically, during the past few decades, Beijing has been undergoing rapid urbanization. Especially since the 1970 s, its urban areas have expanded significantly, and its built-up area increased from 232.13 km^2^ in 1978 to 1268 km^2^ in 2013. The population had reached 21.15 million by 2013^49^. This rapid urbanization is mostly attributable to the concentric expansion of the urban area. Ring roads, which here serve as indicators of urban expansion, were established in different years. They are associated with residential areas of different ages, population densities, and related socioeconomic variables. In this way, urban sprawl from the city center (Forbidden City) has extended outward, forming five concentric areas ringed by roads, which here served as dividers between areas at different stages of urban development. Beijing was here considered a significant opportunity to address the issue of how microbial communities are affected by urban development.

Here, the manner in which microbial communities respond to urban development and the factors driving the composition of microbial communities in urban soil were determined. The relationship between the composition and diversity of the microbial community and urban ring road areas was assessed. It is here hypothesized that changes in urban development may affect the diversity and composition of the soil bacterial community. Specifically, it is here hypothesized that the changes in the bacterial communities were predominantly caused by alterations in the properties of the edaphic environment. The aims of this study were as follows: (i) to identify the dominant bacterial taxa in urban green land at different stages of urban development in Beijing, (ii) to assess the relative abundance and diversity of bacteria in these soils, and (iii) to determine which factors have important effects on the distribution of bacterial communities in urban soil and the most important driving factors determining the composition of the microbial community in urban soil.

## Results

### Physicochemical properties of the soil samples

The physicochemical properties of the soil samples, including the soil pH, moisture, soil bulk density (SBD), total carbon (TC) content, total nitrogen (TN) content, and electric conductivity (EC) are summarized in Table S1. The collected samples had pH values, ranging from 7.25 to 8.46. Most of the urban soil samples were alkalescent or alkaline. However, only 2 samples (both from 2–3 ring road) had pH values below 7.5. Soil total C varied from 11.77 to 53.53 g kg^−1^, soil and total N ranged from 0.99 to 4.25 g kg^−1^. There was no correlation between the SBD and the ring roads of the sampling sites (*r* = 0.273, *P* = 0.069). In contrast, the total soil C (*r* = −0.449, *P* = 0.002), total soil N (*r* = −0.411, *P* = 0.005), moisture (*r* = −0.497, *P* = 0.001), soil EC (*r* = 0.535, *P* < 0.001), and soil pH (*r* = 0.384, *P* = 0.009) were significantly closely correlated with ring roads (Table S2).

### Composition of bacterial communities dominant taxa in urban soils

**Figure 1:**
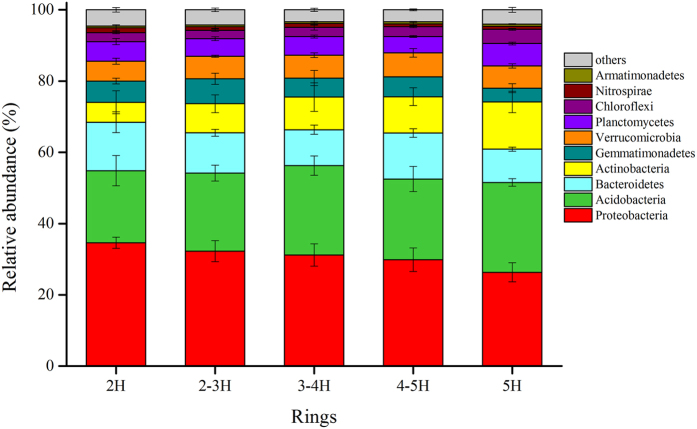
Relative abundance of the dominant bacterial community at the phylum level in samples separated by ring road category. Relative abundances were found to depend on the average relative number of the bacterial sequences of nine samples from each ring. Here “other” means to the taxa with a maximum abundance of <0.5% in any sample.

In total, 1,501,775 quality sequences were obtained from all 45 samples in this study, and the number of sequences varied from 18,780 to 81,399 per sample (mean = 33,373). Of these sequences, 91.04% were classified as bacterial sequences. Bacterial sequences were clustered into OTUs at ≥97% similarity level, 264,564 OTUs were obtained from all of the soil samples, ranging from 7,046–21,388 OTUs per sample. The predominant phyla across all soil samples (relative abundance >5%) were Proteobacteria, Acidobacteria, Bacteroidetes, Actinobacteria, Gemmatimonadetes, and Verrucomicrobia, accounting for more than 82% of the bacterial sequences (Fig. 1). The phyla Planctomycetes, Chloroflexi, Nitrospirae, Armatimonadetes, OD1, Cyanobacteria, TM7, WS3, OP3, Firmicutes, Elusimicrobia, and FBP had a relatively low abundance (relative abundance >0.1%) but were still identified in all of the samples. In addition, 15 fewer phyla were also present in most of the soil samples, and 16 rare phyla were found in several of the samples (Table S3). Proteobacteria and Actinobacteria were the two most abundant phyla in all of the samples, accounting for 43–64% of all bacterial sequences from each ring. The bacterial communities from all areas sampled showed the same dominant taxa but different rare taxa.

Of the main abundant phyla, Proteobacteria (*F*_*4,40*_ = 3.618, *P* = 0.045), Bacteroidetes (*F*_*4,40*_ = 3.828, *P* = 0.039), Actinobacteria (*F*_*4,40*_ = 3.189, *P* = 0.023), Planctomycetes (*F*_*4,40*_ = 4.308, *P* = 0.028), and Chloroflexi (*F*_*4,40*_ = 6.533, *P* = 0.007) showed significant differences in relative abundance among different ring road areas (Fig. S1).

To analyze the influences of location and soil pH on relative abundance of dominant bacterial phylum. Ring road areas were closely correlated with the relative abundance of some phyla (Fig. S2). The relative abundance of Acidobacteria (*r* = 0.331, *P* = 0.026), Actinobacteria (*r* = 0.481, *P* = 0.001), Chloroflexi (*r* = 0.522, *P* < 0.001), Armatimonadetes (*r* = 0.31, *P* = 0.038) were significantly closely correlated with ring roads, and Proteobacteria (*r* = −0.5, *P* < 0.001), Bacteroidetes (*r* = −0.386, *P* = 0.009), Gemmatimonadetes (*r* = −0.391, *P* = 0.008) showed a significant negative correlation with ring roads, and Nitrospirae (*r* = −0.286, *P* = 0.057) showed a marginally significant negative correlation with ring roads.

Soil pH was also closely correlated with the relative abundance of four dominant bacterial phyla. The relative abundances of Acidobacteria (*r* = 0.431, *P* = 0.003), Planctomycetes (*r* = 0.57, *P* < 0.001), and Nitrospirae (*r* = 0.365, *P* = 0.014) were significantly positively correlated with soil pH, while that of Gemmatimonadetes (*r* = −0.391, *P* = 0.008) was significantly negatively correlated with soil pH (Fig. S3).

Results showed that the relative abundance of some phyla, such as Nitrospirae, Gemmatimonadetes, and Acidobacteria, had significant correlations with both ring road location and pH, but other phyla were only significantly correlated to one or the other. The most abundant phyla were Proteobacteria and Actinobacteria; both were only significantly correlated with the ring roads, and neither were correlated with soil pH. The relative abundance of Proteobacteria and Actinobacteria were strongly significantly negatively correlated with each other (*r* = −0.813, *P* < 0.001) (Fig. S4).

### Variations in the diversity of the bacterial community

**Figure 2:**
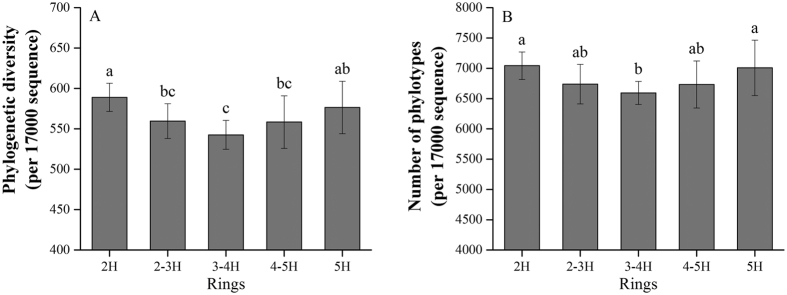
Ring road areas and the (**A**) phylogenetic diversity and (**B**) phylotype richness of soil bacterial OTUs. Diversity indices were calculated using random selections of 17000 sequences per soil sample.

**Figure 3:**
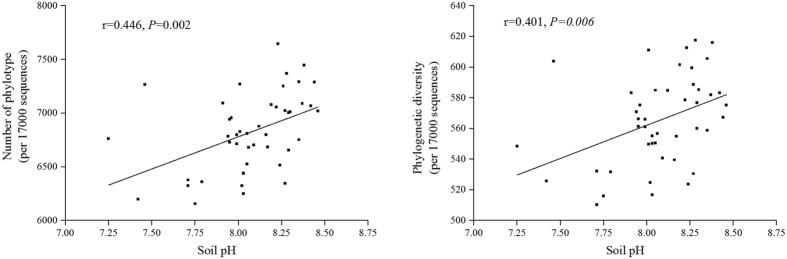
Relationship among soil pH, bacterial phylogenetic diversity, and OTUs phylotype richness.

Both Faith’s phylogenetic diversity (PD) and phylotype richness were used to compare the levels of bacterial diversity. Results are presented in Fig. 2. The diversity of bacterial communities showed high-low-high changes across the ring road area gradient, differing significantly by area (*P* = 0.04; *P* = 0.027, respectively). Samples from 2 H and 5 H showed higher levels of diversity than samples from 3–4 H. Both the phylogenetic diversity and the phylotype richness in 2–3 H and 4–5 H soil samples were toward the middle of the values of all samples collected throughout the five ring road areas. Correlation analysis showed soil pH to be significantly positively correlated with both phylotype richness (*r* = 0.446, *P* = 0.002) and phylogenetic diversity (*r* = 0.401, *P* = 0.006) (Fig. 3). No other soil properties showed any significant relationship to bacterial diversity (Table S4).

### Environmental factors and bacterial community structure

**Figure 4:**
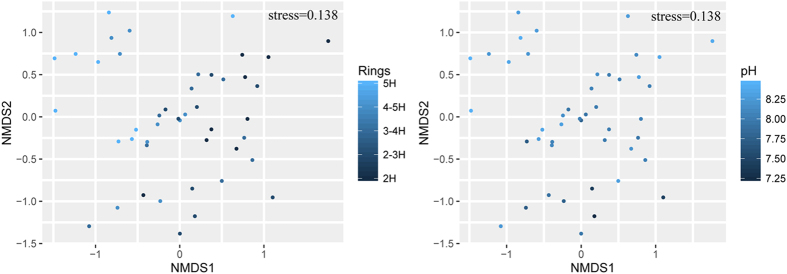
Non-metric multidimensional scaling ordination results showing relationships among urban community composition. Samples are color-coded to indicate (**A**) ring road areas and (**B**) soil pH. Each point represents an individual sample.

Non-metric multidimensional scaling (NMDS) ordinations were performed to assess the microbial community structure of all samples. The NMDS plots indicated that bacterial community structures differed significantly among ring road areas (Mantel test *r* = 0.338, *P* = 0.001) were also strongly influenced by soil pH (Mantel test *r* = 0.285, *P* = 0.001) (Fig. 4). The relationships between the NMDS scores, ring roads, and pH values were evaluated using linear regression, and results showed ring roads to have a significant linear relationship with NMDS1 and soil pH to have a significant linear relationship with NMDS2 (Fig. S5). The changes in bacterial community structures can be attributed to their locations in different road areas across NMDS1 axis and to soil pH along the NMDS2 axis.

### Correlations between environmental parameters and composition of the bacterial community

**Figure 5:**
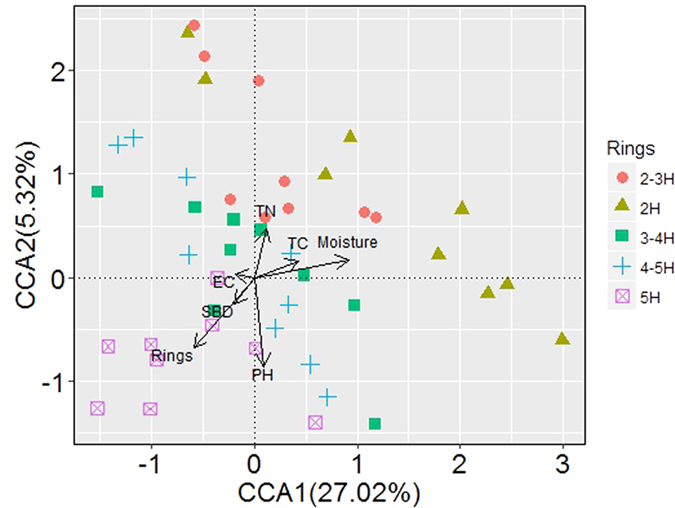
Canonical correspondence analysis (CCA) of sequencing data and environmental factors with symbols coded by ring roads category.

**Figure 6:**
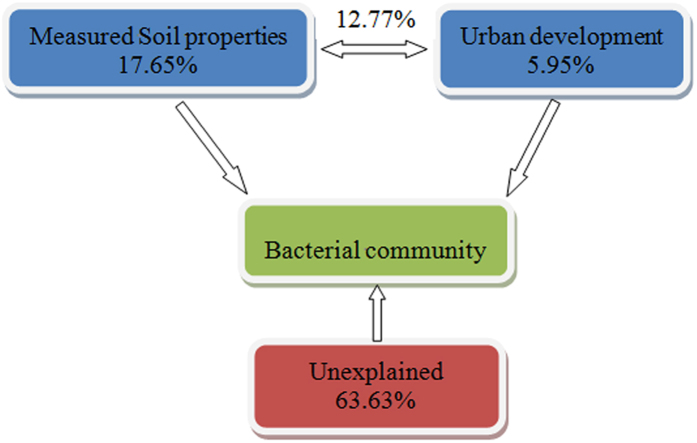
Variance partition analysis of microbial community explained by soil properties and urban development.

CCA was conducted to identify the environmental factors that had the most important effect on bacterial community structure (Fig. 5). Urban ring road area, pH, and moisture were found to be the most important factors (longer arrows) affecting microbial community composition. Variance partitioning analysis was performed to quantify the contributions of soil properties and urban ring roads to the bacterial community variation (Fig. 6). A total of 36.37% of the variation was explained using these environmental variables. Soil properties and urban development independently explained 17.65% and 5.95% of the total bacterial community variance, respectively. Interactions between soil properties and urban development explained 12.77% of the variance.

## Discussion

It is here speculated that urban development can significantly alter the soil microbial community. Results indicated that all the areas studied had the same dominant phyla, but relative abundance differed significantly. The main phyla Proteobacteria, Acidobacteria, Bacteroidetes, Actinobacteria, Gemmatimonadetes, Verrucomicrobia, Planctomycetes, and Chloroflexi, were observed in all soil samples studied here. These results were similar to the findings reported by Xu *et al*. in soil from urban park soil in China^44^. They were also consistent with a study performed in New York City’s Central Park^45^,^50^. These phyla were also found in the Park Grass Experiment^25^. This suggests that the dominant phyla of bacterial communities in urban soil may be similar. Although the main phyla in the communities are constant, relative abundances vary significantly among samples. Several rare phyla were only observed in some samples, indicating that rare phyla are crucial to the study of composition of the soil bacterial community in soil at different stages of urban development.

Urbanization has led to homogenization of plant and animal, and has caused the extinction of some native species^5^,^7^. Urbanization also increases the non-native plant and animal populations^2^,^51^. For soil bacteria, it is not clear how urbanization might affect microbial communities. Xu *et al*. suggested that urbanization factors have no significant correlation with α-diversity in urban parks^44^. However, in the current work, results showed that soil bacterial diversity had significant differences across urban ring road areas. Areas 2 H and 5 H showed more diversity than 3–4 H, indicating that urban construction may influence the soil bacterial diversity but does not necessarily reduce the diversity of soil bacteria. The diversity of the bacteria found in the global samples and in New York City’s Central Park was similar^45^. Reese *et al*. also found the diversity of bacteria in the medians to be similar to that of Central Park^50^. It should be noted that the samples in other studies^44^,^45^,^50^ were collected from urban parks subject to no direct disturbances from human activities, suggesting that the bacterial communities of urban park soil microbial communities might not respond to direct disturbances by urban development. More studies should be performed to confirm this.

The results reported here show that soil moisture and TC changed considerably, and the strongest relationship among EC, moisture, and TC in different ring road areas. In this study, soil pH was found to differ significantly among ring road areas and to have a significantly close correlation with urban ring roads (*P* < 0.05). This indicates that urban development can affect the properties of the edaphic environment. Shifts in bacterial communities can be expected to be associated with differences in urban development that are directly or indirectly related to the effects of changes in urban development edaphic properties.

In this study, results showed that some phyla display correlations to variations in pH. The patterns of some specific phyla across this pH gradient are similar to their pH responses observed in other studies. For instance, the relative abundance of Planctomycetes was here shown to decrease with increasing pH, as in other studies^44^. The relative abundance of Acidobacteria has been shown to increase with decreasing pH^20^,^22^, in contrast to the present results. Here, the two most abundant phyla, Proteobacteria and Actinobacteria, showed no significant correlation with pH value, and they were negatively correlated with each other. It is here speculated that Proteobacteria and Actinobacteria are less sensitive to pH in urban soil than bacteria from other phyla are. This result was consistent with results reported by Xu *et al*.^44^. However, it was here observed that Proteobacteria and Actinobacteria were significant closely correlated with urban ring roads. This means that, in order to study the effect of urban development on the composition of soil bacterial communities, the changes in the abundance of Proteobacteria and Actinobacteria should be considered. Several highly abundant taxa were also observed to have positive and negative relationships with the ring roads. This indicated that stages of urban development also have a strong influence on relative abundance of bacterial phyla. In general, pH and ring roads affect the composition of the soil bacterial community by changing the abundances of different taxa.

Notably, the present study demonstrates that bacterial diversity and community composition are mainly correlated with soil pH. As shown in many previous studies, soil pH is key to shaping the composition of soil bacterial community^21^,^52^. Soil pH also has a strong correlation with microbial diversity^19^,^24^. Soil pH is considered one of the best predictors of variation in microbial diversity^20^,^21^,^22^,^25^. In this way, soil pH is a universally good predictor of bacterial distribution patterns^53^, but it is not the only one. For example, Liu *et al*. reported soil C content to be significantly closely correlated with bacterial diversity and bacterial community structure^53^. Precipitation and soil NH_4_^+^ are shown to have considerable influence on bacterial communities at depth of 0–5 cm^36^. Temperature and NH_4_^+^–N may be two of the key impact factors that shape microbial community structure^54^. History of land-use was a great determinant of the composition of microbial communities^55^. Plant diversity was found to be crucial to shaping the microbial community and its diversity^56^. For this reason, it is here speculated that different factors can influence microbial communities according to different ecosystems and environmental conditions. Factors, such as soil metal concentration, polycyclic aromatic hydrocarbons (PAHs) content, temperature, nutrient availability, and texture should be considered in subsequent assessments of urban soil microbial communities.

Both the CCA and relationship analysis showed that ring roads and soil microbial diversity to be significantly correlated (*P* < 0.01). Ring roads may be important factors that alter soil bacterial community structure and diversity during urban development in Beijing. Urbanization processes were found to exert some influence on soil bacterial community composition, and urbanization indexes were found to be significant predicting factors^44^. The ring roads of Beijing were completed in different years, and urban development from the city center to the outskirts can be measured using the ring roads. The gradient of urban ring roads is a suitable way to represent the stages of urban development. For this reason, it is here speculated that ring roads (representing different stages of urban development) are also important driving factors of bacterial communities in the urban soil in Beijing. Further, microbial communities in disturbed soil have been found to return to their native state over time^55^. Soil bacterial communities can change in response to the disturbance caused by urban construction; however, the order of succession is unclear.

Urban development was found to independently explain 5.95% of the variation in the soil bacterial community and soil properties to explain and 17.65%. Soil properties are dominant factors that influence composition of the soil bacterial community during urban development. Results here showed that interactions between soil properties and urban development are also an import part of shaping the composition of soil bacterial communities. As reported by Xu *et al*., however, a large part of the variation observed in such communities has remained unexplained^44^. Other factors, such as soil metal concentration, plants, population density, and gross domestic product per square kilometer (GDP km^−2^) should be considered in further studies of urban soil microbes.

In conclusion, this study indicated that the dominant bacterial phyla in all of the samples in different ring roads of Beijing are Proteobacteria, Acidobacteria, Bacteroidetes, Actinobacteria, Gemmatimonadetes, Verrucomicrobia, Planctomycetes, and Chloroflexi. Soil pH is an important driving factor that shapes soil bacterial diversity and community composition among different ring road areas. Urban ring roads are also another principal factor contributing to the variation in the composition of soil bacterial communities, and a relatively high diversity of the bacterial community was observed at 2 H and 5 H. About 63.63% of the total variance could not be explained by these environmental factors, indicating that urban development has complex effects on soil microbial communities in Beijing and cannot be easily predicted using common factors.

## Materials and Methods

### Site and sampling

**Figure 7:**
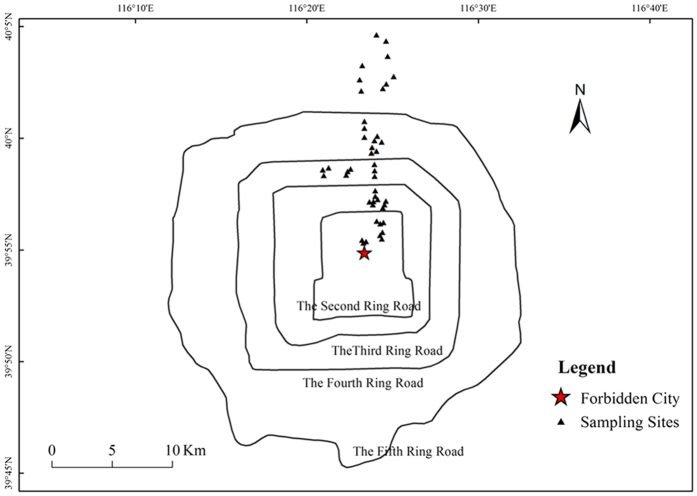
Sampling sites in the urban area of Beijing(created using ArcGIS 10.1 http://www.esri.com/ software).

The metropolitan city of Beijing was selected as the study area (39.4°N– 41.6°N, 115.7°E– 117.4°E). Beijing is the capital of China, and it has a 3,000-year history of urban construction, and more than 800 years of history as the political and cultural center of China. Soil samples were collected from the center to northern parts of Beijing’s built-up area in August 2014. A total of five sites were selected, one in an area ringed by the second ring roads (2 H), one in the area between the second and third ring roads (2–3 H), one in the area between the third and fourth ring roads (3–4 H), one in the area between the fourth and fifth ring roads (4–5 H), one in area outside of the fifth ring road (5 H) (Fig. 7). A map of the sampling sites was drawn using ArcGIS 10.1 (http://www.esri.com/). At each site, soil samples were collected from 9 plots (10 m × 10 m) as nine independent replicates. In each plot, samples of the soil top layer (10 cm × 10 cm in area, and 0–10 cm depth directly below the litter layer) were collected at five random points using a sterile blade. After collection, the soil samples were composited together as a single sample and sieved through a 2 mm mesh to thoroughly homogenize and remove the roots, plant residues and stones. All samples were divided into two subsamples. One subsample was air-dried to determine the physical and chemical properties of the soil, and one was stored at −80 °C for DNA extraction.

### Soil property measurements

The soil moisture of each sample was measured gravimetrically, after the sample had been dried in an oven at 105 °C for 12 h. Soil bulk density (SBD) was determined from an undisturbed soil core. The soil pH was measured by pH meter (FE20-FiveEasyTM pH, Mettler Toledo, German) after shaking a soil water (1:2.5 wt/vol) suspension for 30 min. The electrical conductivity (EC) of each soil sample was tested using the electromagnetic conductivity meter as soil:water ratio of 1:5 (1:5 wt/vol). The soil total carbon (TC) and total nitrogen (TN) were determined using an elemental analyzer (Costech ECS 4012, Italy)

### DNA extraction, PCR and high-throughput sequencing

For each soil sample, microbial DNA was extracted from 0.5 g wet soil using a FastDNA^®^ SPIN Kit for soil (MP Biomedicals, Santa Ana, CA, U.S.) according to the manufacturer’s instructions. The extracted soil DNA was dissolved in 50 μl TE buffer, and DNA concentrations were measured using the NanoDrop^TM^ 2000 spectrophotometer (Thermo Scientific, U.S.), and stored at −20 °C until use. Bacterial DNA was sequenced for all sites targeting the V4 region of the bacterial 16 S rRNA gene, PCR amplification by the 515 F: GTGCCAGCMGCCGCGGTAA and 806 R: GGACTACHVGGGTWTCTAAT primers^57^. The paired-end sequencing was done using the Illumina MiSeq platform (Illumina Inc., San Diego, CA, U.S.) at the Beijing Genome Institute (BGI).

### Sequence analysis

Sequence data were processed using the Quantitative Insights Into Microbial Ecology (QIIME) software package^58^. Reads were filtered using QIIME quality filters to remove low-quality reads. Then qualified sequences were clustered into operational taxonomic units (OTUs) at a 97% similarity level. The most abundant sequence in each OUT was selected as representative of that OUT and aligned using PyNAST^59^. The taxonomic identity of each OTU was determined using an RDP classifier^60^. In order to ensure that sampling depth would remain the same across all samples, samples were rarified to 17,000 sequences each for analysis.

### Data analyses

One goal of this study was to assess the correlations between the relative abundance of bacterial phyla, bacterial diversity, and soil properties, using linear regression analyses. For alpha diversity, Phylogenetic diversity and phylotype richness were chosen to asses the internal (within-sample) complexity of individual microbial population. Phylogenetic diversity was estimated using Faith’s index, which incorporates the phylogenetic breadth across taxonomic levels^61^,^62^. Here, one-way ANOVA to determine whether relative abundance of the most abundant phyla and bacterial diversity differed among rings. For beta diversity, Bray-Curtis distance was used to measure the differences in overall community composition between each pair of samples. The Bray-Curtis index was used to indicate community structure because it is sensitive to the difference in abundance observed between the same taxa across pairs of samples. Non-metric multidimensional scaling (NMDS) was conducted based on Bray–Curtis distances for bacterial community composition dissimilarity analysis. The relationship between community composition variation and environmental factors was assessed by fitting the ordination scores to soil parameters and urban ring roads. Canonical correspondence analysis (CCA) was performed to identify the abiotic factors most important to shaping variation in bacterial community structure. Variation partitioning analysis was used to evaluate the influence of soil parameters and urban rings on bacterial community structure. The R project for statistical analysis package was used for all statistical analyses that involved different packages^63^.

## Additional Information

**How to cite this article**: Yan, B. *et al*. Urban-development-induced Changes in the Diversity and Composition of the Soil Bacterial Community in Beijing. *Sci. Rep.* **6**, 38811; doi: 10.1038/srep38811 (2016).

**Publisher's note:** Springer Nature remains neutral with regard to jurisdictional claims in published maps and institutional affiliations.

## Supplementary Material

Supplementary Information
