# Supplementary material for: Urban-development-induced Changes in the Diversity and Composition of the Soil Bacterial Community in Beijing
Source: Sci Rep. 2016 Dec 9;6:38811. doi: 10.1038/srep38811 (PMC5146926; doi:10.1038/srep38811)
Supplement: Supplementary Information [file srep38811-s1.pdf]

Supplementary information

Urban-development-induced Changes in the Diversity and Composition of the Soil Bacterial Community in Beijing

Bing Yan[1,2], Junsheng Li[2*], Nengwen Xiao[2], Yue Qi[1,2], Gang Fu[1,2], Gaohui Liu[2], Mengping Qiao[3]

[1]College of Water Sciences, Beijing Normal University, Beijing, China

[2]Chinese Research Academy of Environmental Sciences, Beijing, China

[3]School of Environment and Natural Resources, Renmin University of China, Beijing, China

[*]Correspondence and requests for materials should be addressed to J.L. (lijsh@craes.org.cn)

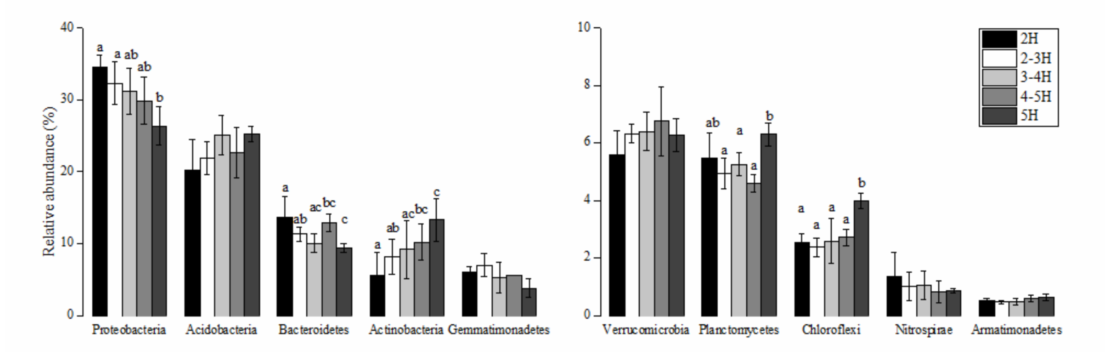

Figure. S1 Relative abundance (means ±SD) of the most abundant phyla in different ring areas. Different letters denote significant differences between groups.

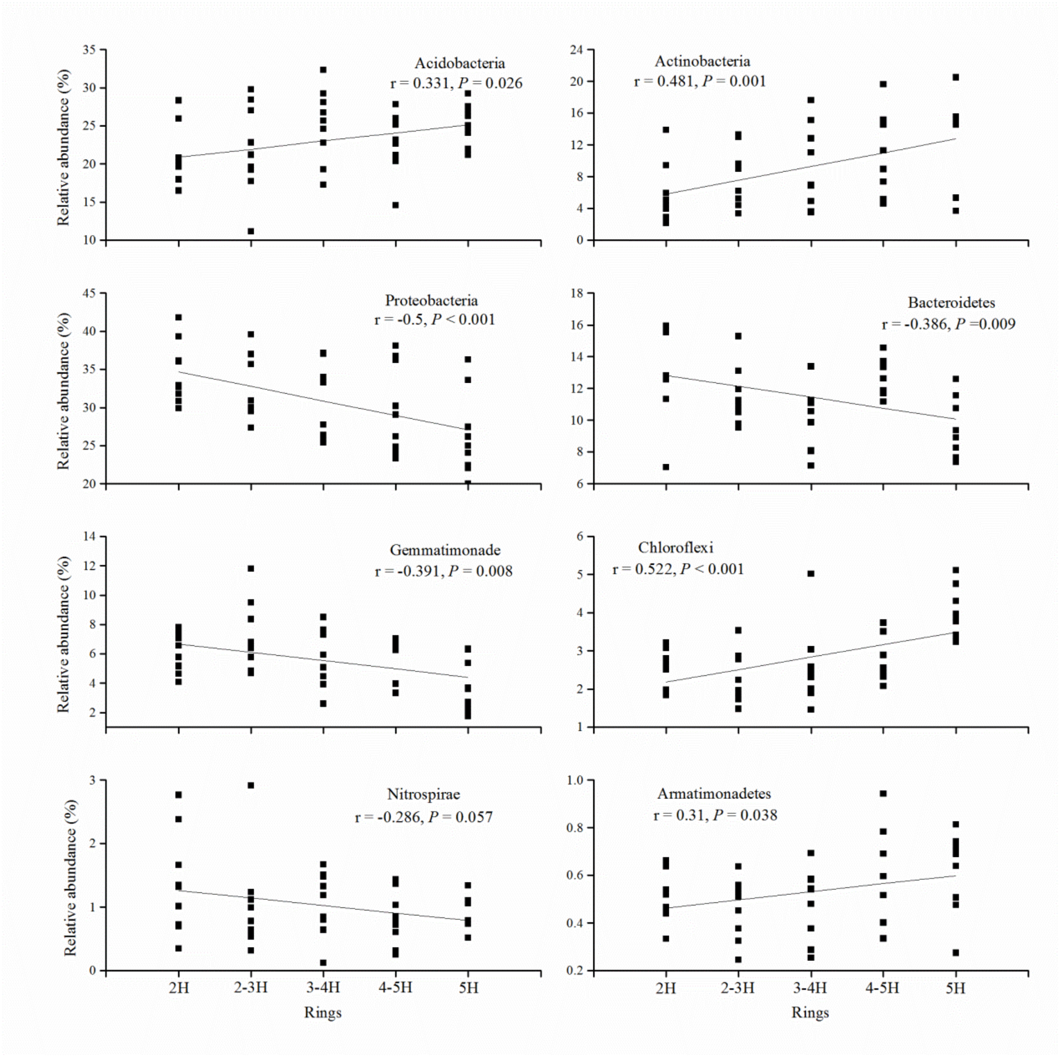

Figure. S2 Relationship between relative abundances of dominant bacterial phyla and ring roads.

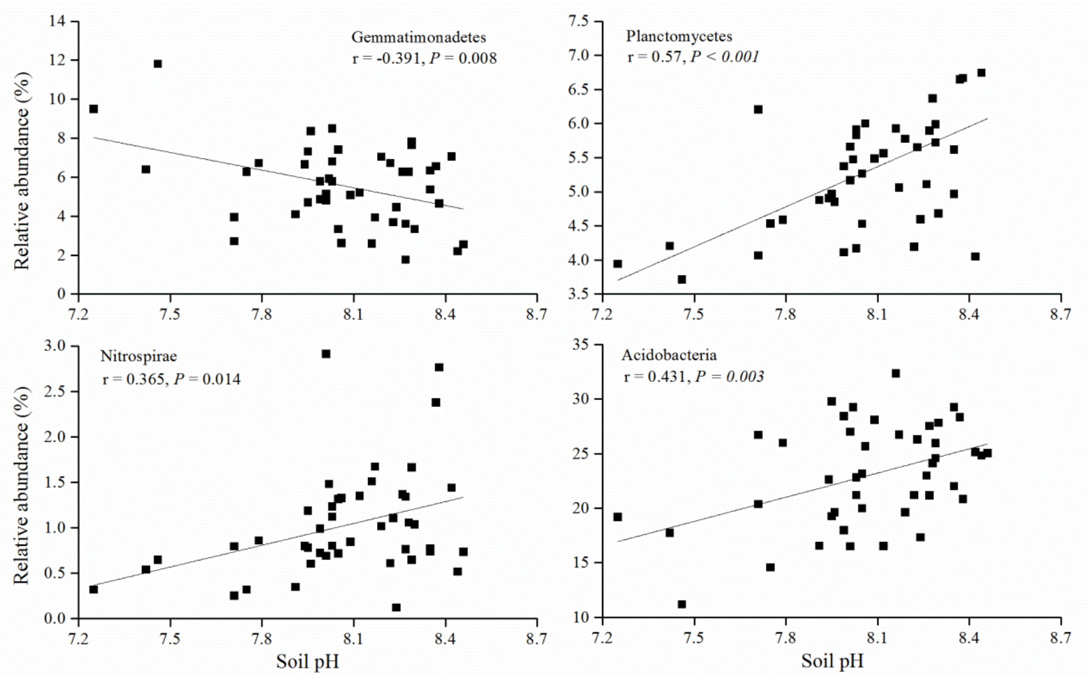

Figure. S3 Relationship among relative abundances of dominant bacterial phyla and soil pH.

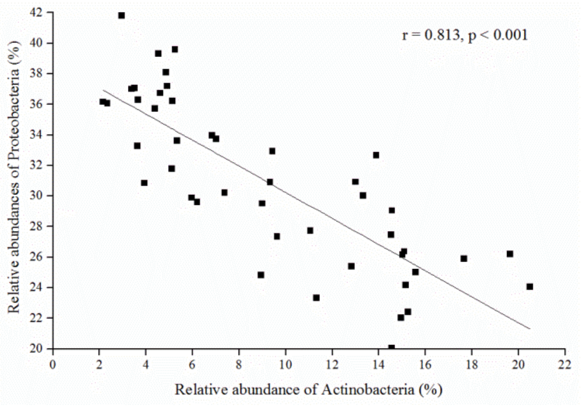

Figure. S4 Relationship between the relative abundance of Proteobacteria and Actinobacteria.

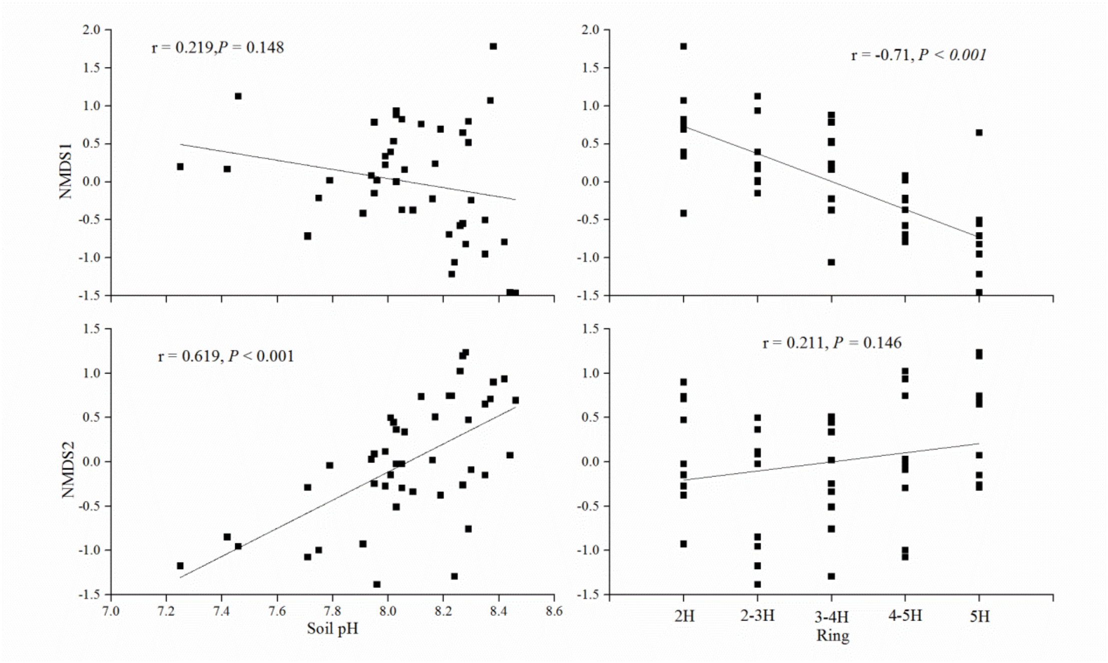

Figure. S5 Pairwise regression of NMDS scores and soil pH value for urban ring road areas.

Table S1 Soil physical and chemical properties used for this study

Sample	pH	Moisture(%)	EC (ds m^{-1})	SBD (g cm^{-3})	Total C (g kg^{-1})	Total N (g kg^{-1})
2H	8.15±0.17ab	18.42±6.19a	1.15±0.32b	1.325±0.11a	31.67±9.36a	1.77±0.80a
2-3H	7.88±0.31c	15.42±3.38ab	1.37±0.32b	1.334±0.12a	29.44±9.12a	1.45±0.38ab
3-4H	8.11±0.11ab	11.72±4.79bc	1.43±0.43b	1.360±0.09a	31.38±17.69ab	1.42±0.78ab
4-5H	8.10±0.25b	12.43±2.8bc	2.69±1.17a	1.238±0.42a	24.92±6.66ab	1.20±0.62ab
5H	8.31±0.11a	9.27±5.86c	2.24±1.18a	1.434±0.13a	19.79±4.30b	1.07±0.40b

Means of nine replicates per sites are presented (with standard deviation).
Different letters in the same column indicate significant difference between sites (P < 0.05).

Table S2 Spearman's correlations (r) between urban ring roads and soil characteristics.

	r	p
pH	0.384	0.009
EC	0.535	<0.001
SBD	0.273	0.069
TC	-0.449	0.002
TN	-0.411	0.005
Moisture	-0.497	0.001

TC, total carbon; TN, total nitrogen; SBD, soil bulk density; EC, electrical conductivity.

Table S3 Classified phyla from all the soil samples collected from Beijing.

predominant phyla (relative abundance>5%)	low abundant phyla (relative abundance >0.1%)	fewer phyla (present in most of the samples)	rare phyla (present in several of the samples)
p__Proteobacteria	p__Planctomycetes	p__Spirochaetes	p__AC1
p__Acidobacteria	p__Chloroflexi	p__TM6	p__AD3
p__Bacteroidetes	p__Nitrospirae	p__Tenericutes	p__AncK6
p__Actinobacteria	p__Armatimonadetes	p__WPS-2	p__Deferribacteres
p__Gemmatimonadetes	p__OD1	p__WS2	p__FCPU426
p__Verrucomicrobia	p__Cyanobacteria	p__Thermi	p__Fusobacteria
	p__TM7	p__NKB19	p__GN04
	p__WS3	p__OP11	p__GOUTA4
	p__OP3	p__BHI80-139	p__Kazan-3B-28
	p__Firmicutes	p__BRC1	p__Lentisphaerae
	p__Elusimicrobia	p__Chlamydiae	p__NC10
	p__FBP	p__Fibrobacteres	p__PAUC34f
		p__GN02	p__SBR1093
		p__GAL15	p__SR1
		p__MVP-21	p__ZB3
			p__Caldithrix

Table S4 Pearson correlations(r) between bacterial diversity (Faith's PD, OTUs) and soil and site characteristics.

r	Rings	TC	TN	pH	SBD	Moisture	EC
PD	-0.128	-0.078	-0.042	0.401**	-0.017	0.199	-0.219
OTUs	0.03	-0.099	-0.092	0.446**	0.028	0.01	-0.25

TC, total carbon; TN, total nitrogen; SBD, soil bulk density; EC, electrical conductivity; **P<0.01.
